# Topological properties of some sequences defined over 2-normed spaces

**DOI:** 10.1186/s40064-016-3646-7

**Published:** 2016-11-14

**Authors:** Hemen Dutta, Adem Kilicman, Omer Altun

**Affiliations:** 1 Department of Mathematics, Gauhati University, Guwahati, 781014 India; 2 Department of Mathematics and Institute for Mathematical Research, University Putra Malaysia (UPM), 43400 Serdang, Selangor Malaysia

**Keywords:** Orlicz function, 2-Norm, Paranormed space, Completeness, Solidity, 40A35, 40A05, 40C05, 40D05, 46A45

## Abstract

The paper investigates some classes of real number sequences over 2-normed spaces defined by means of Orlicz functions, a bounded sequence of strictly positive real numbers, a multiplier and a normal paranormed sequence space. Relevant properties of such classes have been investigated. Moreover, relationships among different such classes of sequences have also been studied under various parameters and conditions. Finally, the spaces are investigated for some other useful properties. The conclusion section provides many interesting facts for further research.

## Background

The work of this paper is related to functional analytic study of Orlicz sequence space as well as composite Orlicz sequence spaces of real number over 2-normed spaces. From functional analytic point of view, the Orlicz sequence spaces are the special cases of Orlicz spaces studied in Krasnoselskii and Rutisky (1961). Lindenstrauss and Tzafriri (1971) first investigated Orlicz sequence spaces in detail with certain aims in Banach space theory.

An Orlicz function is a function *M*: [0, ∞) → [0, ∞), which is continuous, nondecreasing and convex with *M*(0) = 0, *M*(*x*) > 0 for *x* > 0, and *M*(*x*) → ∞ as *x* → ∞.

An Orlicz function *M* is said to satisfy Δ_2_-condition for all values of *x*, if there exists a constant *L* > 0, such that *M*(2*x*) ≤ *LM*(*x*) for all *x* ≥ 0. The Δ_2_-condition implies $M(lx) \le Ll^{{\log_{2} L}} M(x)$ for all *x* > 0, *l* > 1. Also an Orlicz function satisfies the inequality *M*(*λx*) ≤ *λM*(*x*) for all *λ* with 0 < *λ* < 1 (Rao and Ren 1991).

If convexity of Orlicz function *M* is replaced by *M*(*x* + *y*) ≤ *M*(*x*) + *M*(*y*), then the function reduces to a modulus function. For more details about this function and its subsequent use, one may refer to Krasnoselskii and Rutisky (1961), Kamthan and Gupta (1981), Rao and Ren (1991), Ruckle (1973), Maddox (1986), Ghosh and Srivastava (1999), Srivastava and Kumar (2010), Altin (2009), Debnath and Saha (2015), and many others.

Lindenstrauss and Tzafriri (1971) used the idea of Orlicz function to construct the sequence space

$$ \ell_{M} = \left\{ {x \in w: \sum\limits_{k = 1}^{\infty } {\left( {M\left( {\frac{{|x_{k} |}}{\rho }} \right)} \right)} < \infty , \quad {\text{for}}\;{\text{some}}\; \;\rho > 0} \right\}. $$

The space *ℓ*_*M*_ with the norm

$$ \left\| x \right\| = inf\left\{ {\rho > 0: \sum\limits_{k = 1}^{\infty } \left( {M\left( {\frac{{|x_{k} |}}{\rho }} \right)} \right) \le 1} \right\} $$

becomes a Banach space which is called an Orlicz sequence space, where *w* is the family of real or complex sequences. Güngör et al. (2004), Esi et al. (2004), Nuray and Gülcü (1995), Dutta and Bilgin (2011), Mursaleen et al. (2001), Ahmad and Bataineh (2001), Bektas and Altin (2003), Parashar and Choudhary (1994), Savas (2010), Isik (2012), Dutta and Başar (2011), Karakaya and Dutta (2011), Tripathy and Dutta (2012), Dutta and Jebril (2013), Khan and Tabassum (2011), Debnath and Debnath (2014), and many others have used Orlicz function to construct several new sequence spaces.

Let *X* be a real linear space with dimension >1 and let ‖.,.‖ be a real-valued function on *X* × *X* satisfying the following conditions:

1. ‖*x*, *y*‖ = 0 if and only if *x* and *y* are linear dependent;
2. ‖*x*, *y*‖ = ‖*y*, *x*‖;
3. ‖*αx*, *y*‖ = |*α*|‖*x*, *y*‖ for any real number *α*;
4. ‖*x*, *y* + *z*‖ ≤ ‖*x*, *y*‖ + ‖*x*, *z*‖.

Then ‖.,.‖ is called a 2-norm on *X* and (*X*, ‖.,.‖) is called a linear 2-normed space (Gähler 1965). Some of the basic properties of the 2-norms includes that they are non-negative, and ‖*x*, *y* + *αx*‖ = ‖*x*, *y*‖ for every *x*, *y* ∊ *X* and any real number *α*.

A sequence {*x*_*n*_} in a linear 2-normed space (*X*, ‖.,.‖) is called a Cauchy sequence if lim_*n,m*→∞_‖*x*_*n*_ − *x*_*m*_, *z*‖ = 0 for all *z* ∊ *X*. A sequence {*x*_*n*_} in a linear 2-normed space (*X*, ‖.,.‖) is called a convergent sequence if there is an *x* ∊ *X* such that lim_*n*→∞_‖*x*_*n*_ − *x*, *z*‖ = 0 for all *z* ∊ *X*. A linear 2-normed space in which every Cauchy sequence is a convergent sequence is called a 2-Banach space.

The concept of 2-inner product spaces is closely related to linear 2-normed space. For a real linear space *X* of dimension *d* > 1, let 〈.,.|.〉 be a real-valued function on *X* × *X* × *X* which satisfies the following conditions:

1. 〈*x*, *x*|*z*〉 ≥ 0; 〈*x*, *x*|*z*〉 = 0 if and only if *x* and *z* are linearly dependent;
2. 〈*x*, *x*|*z*〉 = 〈*z*, *z*|*x*〉;
3. 〈*x*, *y*|*z*〉 = 〈*y*, *x*|*z*〉;
4. 〈*αx*, *y*|*z*〉 = *α*〈*x*, *y*|*z*〉 for any real number *α*;
5. 〈*x* + *x* ^/^, *y*|*z*〉 = 〈*x*, *y*|*z*〉 + 〈*x* ^/^, *y*|*z*〉.

Then 〈.,.|.〉 is called a 2-inner product on *X* and (*X*, 〈.,.|.〉) is called a 2-inner product space. In Diminnie et al. (1973), it is shown that $\left\| {x,z} \right\| = \left\langle {x,x|z} \right\rangle^{{\frac{1}{2}}}$ is a 2-norm on (*X*, ‖.,.‖). Hence 2-inner product spaces are 2-normed spaces.

The details about above and associated notions and results, we refer to the book by Freese and Cho (2001). Savas (2010) and Dutta (2010) can be seen for some use of the 2-norm structure in construction of sequence spaces.

Let *P* be a subset of the set of all scalar valued sequences *w*. Now we recall the following notions.

A scalar valued paranormed (Maddox 1970) sequence space (*P*, *g*_*P*_), where *g*_*P*_ is a paranorm on *P* is called monotone paranormed space if *x* = (*x*_*k*_) ∊ *P*, *y* = (*y*_*k*_) ∊ *P* and |*x*_*k*_| ≤ |*y*_*k*_| for all *k* implies *g*_*P*_(*x*) ≤ *g*_*P*_(*y*).

*P* is called normal or solid if *y* = (*y*_*k*_) ∊ *P* whenever |*y*_*i*_| ≤ |*x*_*i*_|, *i* ≥ 1 for some *x* = (*x*_*k*_) ∊ *P*.

A sequence space *P* with linear topology is called a *K*-space provided each of the maps *p*_*i*_: *P* → *C*, *p*_*i*_(*x*) = *x*_*i*_ is continuous, *i* ≥ 1.

A sequence space *P* is said to be symmetric if (*X*_*π*(*k*)_) ∊ *P* whenever (*X*_*k*_) ∊ *P*, where *π* is permutation of ${\mathbb{N}}$.

A sequence space *P* is said to be convergence free if (*X*_*k*_) ∊ *P* when (*Y*_*k*_) ∊ *P* and *Y*_*k*_ = 0 implies *X*_*k*_ = 0.

Let (*P*, *g*_*P*_) be a paranormed space and (*a* ^*n*^) ⊂ *P*, where $a^{n} = \left( {a_{k}^{n} } \right)$. If $a_{k}^{n} \to 0$ as *n* → ∞ for each *k* implies *g*_*P*_(*a* ^*n*^) → 0 as *n* → ∞, then we say that the co-ordinate wise convergence implies convergence in *g*_*P*_, e.g., *c*_0_, *ℓ*_1_, *ℓ*_∞_, etc.

The following inequalities (Maddox 1970) will be used throughout the paper.

### **Proposition 1**

*Let* (*p*_*k*_) *be a bounded sequence of strictly positive real numbers with* 0 < *p*_*k*_ ≤ sup *p*_*k*_ = *H*, *D* = max(1, 2^*H*−1^). *Then*

(i) $$ |a_{k} + b_{k} |^{{p_{k} }} \le D\{ |a_{k} |^{{p_{k} }} + |b_{k} |^{{p_{k} }} \} $$;
(ii) $$ |\lambda |^{{p_{k} }} \le max(1,[\lambda ]^{H} ) $$

## The new class *F*(‖.,.‖, *M*, *p*, *s*) and some other classes

In this section, we construct the new sets to be investigated and give a few descriptions of such sets along with intended aims for results concerning the sets and their possible extensions and derivatives.

Let (*F*, *g*_*F*_) be a normal paranormed sequence space with paranorm *g*_*F*_ which satisfies the following properties:

(i) *g*_*F*_ is a monotone paranorm;
(ii) coordinate wise convergence implies convergence in paranorm *g*_*F*_, which implies that for each $(X^{n} ) = (X_{k}^{n} ) \in F, n, k \in {\mathbb{N}}$,
  $$ X_{k}^{n} \to 0 \quad {\text{as}}\; n \to \infty \quad ({\text{for}}\;{\text{each}}\; k) \Rightarrow g_{F} (X^{n} ) \to 0 \quad {\text{as}}\; n \to \infty $$

Let *M* be a Orlicz function and (*N*, ‖.,.‖) be a 2-normed space. We now define the new class of sequences as follows for every *z* ∊ *N*:

$$ F\left( {\left\| {.,.} \right\|, M, p, s} \right) = \left\{ {X = (X_{k} ):X_{k} \in N,\left( {k^{ - s} \left[ {M\left( {\frac{{\left\| {X_{k} ,Z} \right\|}}{\rho }} \right)} \right]^{{p_{k} }} } \right) \in F, \quad{\text{for}}\,{\text{some}}\, \rho > 0} \right\} $$

where *s* ≥ 0 and {*p*_*k*_} is a bounded sequence of strictly +ve real numbers with inf *p*_*k*_ > 0.

This class give rises different other classes of sequences as follows:

$$ F\left( {\left\| {.,.} \right\|, M^{r} , p, s} \right) = \left\{ {X = (X_{k} ):X_{k} \in N,\left( {k^{ - s} \left[ {M^{r} \left( {\frac{{\left\| {X_{k} ,Z} \right\|}}{\rho }} \right)} \right]^{{p_{k} }} } \right) \in F,\quad {\text{for}}\,{\text{some}}\, \rho > 0} \right\}, $$

where *r* is any positive integer.

$$ F\left( {\left\| {.,.} \right\|,M,s} \right) = \left\{ {X = (X_{k} ):X_{k} \in N,\left( {k^{ - s} \left[ {M\left( {\frac{{\left\| {X_{k} ,Z} \right\|}}{\rho }} \right)} \right]} \right) \in F, \quad{\text{for}}\,{\text{some}}\, \rho > 0} \right\} $$

$$ F\left( {\left\| {.,.} \right\|,p,s} \right) = \left\{ {X = (X_{k} ):X_{k} \in N,\left( {k^{ - s} \left[ {\left( {\frac{{\left\| {X_{k} ,Z} \right\|}}{\rho }} \right)} \right]^{{p_{k} }} } \right) \in F,\quad{\text{for}}\,{\text{some}}\; \,\rho > 0} \right\} $$

and so on.

We define a function on $F\left( {\left\| {.,.} \right\|,M,p,s} \right)$ as follows which is proved to be a paranorm in the next section:

- For *X* = (*X*_*k*_)∊ *F*(‖.,.‖, *M*, *p*, *s*) and *Z* ∊ *N*,
  1 $$ g\left( X \right) = \inf \left\{ {\rho^{{\frac{{p_{k} }}{T}}} > 0:\,\left[ {\,g_{F} \left( {k^{ - s} \left[ {M\left( {\frac{{\left\| {X_{k} ,Z} \right\|}}{\rho }} \right)} \right]^{{p_{k} }} } \right)} \right]^{{\frac{1}{T}}} \le 1,\quad k = 1,2, \ldots } \right\}, $$

where *T* = max(1, *H*), *H* = sup_*k*_ *p*_*k*_ < ∞ and inf *p*_*k*_ > 0.

The above classes of sequences of real numbers give rise to many well known sequence spaces on specifying the space *F*, the Orlicz function *M*, the bounded sequence {*p*_*k*_} of positive real numbers, *s* ≥ 0 and the base space (*N*, ‖.,.‖). Further, we can derive several other similar classes for study. The main results of the paper are obtained using the properties of Orlicz functions, 2-norm spaces and most importantly that are of normal paranormed spaces with monotone paranorm and coordinate wise convergence property. One may find it interesting and useful to study further the sets for several other algebraic and topological properties as well as convergence and completeness related and geometric properties. The last few results also hint for several other possible rich property of the sets.

## Main results

In this section, we first examine the linearity of the sets defined above. Then the sets will be investigated for completeness under a suitably defined paranorm. Further, the sets will be examined for *K*-space property. The next few results will be given for the set *F*(‖.,.‖, *M*, *p*, *s*) only as for other sets the proofs can be obtained applying similar arguments.

### **Theorem 1**

*The set F*(‖.,.‖, *M*, *p*, *s*) *is linear over the set of real numbers* ${\mathbb{R}}$.

### *Proof*

Let *X* = (*X*_*k*_), *Y* = (*Y*_*k*_) ∊ *F*(‖.,.‖, *M*, *p*, *s*) and $\alpha , \beta \in {\mathbb{R}}$. Then there exist some positive numbers *ρ*_1_ and *ρ*_2_ such that for every *z* ∊ *N*

$$ \left( {k^{ - s} \left[ {M\left( {\frac{{\left\| {X_{k} ,Z)} \right\|}}{{\rho_{1} }}} \right)} \right]^{{p_{k} }} } \right) \in F\quad {\text{and}}\quad\left( {k^{ - s} \left[ {M\left( {\frac{{\left\| {X_{k} ,Z)} \right\|}}{{\rho_{2} }}} \right)} \right]^{{p_{k} }} } \right) \in F $$

Let us choose *ρ* = max{2|*α*|*ρ*_1_, 2|*β*|*ρ*_2_} so that

$$ \begin{aligned} & k^{ - s} \left[ {M\left( {\frac{{\left\| {\alpha X_{k} + \beta Y_{k} ,Z} \right\|}}{\rho }} \right)} \right]^{{p_{k} }} \\ & \quad \le k^{ - s} \left[ {M\left( {\frac{{\left\| {\alpha X_{k} ,Z} \right\| + \left\| {\beta Y_{k} ,Z} \right\|}}{\rho }} \right)} \right]^{{p_{k} }} \\ & \quad = k^{ - s} \left[ {M\left( {|\alpha |\frac{{\left\| {X_{k} ,Z} \right\|}}{\rho } + |\beta |\frac{{\left\| {Y_{k} ,Z} \right\|}}{\rho }} \right)} \right]^{{p_{k} }} \\ & \quad \le k^{ - s} \frac{1}{{2^{{p_{k} }} }}\left[ {M\left( {\frac{{\left\| {X_{k} ,Z} \right\|}}{{\rho_{1} }}} \right) + M\left( {\frac{{\left\| {Y_{k} ,Z} \right\|}}{{\rho_{2} }}} \right)} \right]^{{p_{k} }} \\ & \quad < k^{ - s} \left[ {M\left( {\frac{{\left\| {X_{k} ,Z} \right\|}}{{\rho_{1} }}} \right) + M\left( {\frac{{\left\| {Y_{k} ,Z} \right\|}}{{\rho_{2} }}} \right)} \right]^{{p_{k} }} \\ & \quad \le Dk^{ - s} \left[ {M\left( {\frac{{\left\| {X_{k} ,Z} \right\|}}{{\rho_{1} }}} \right)} \right]^{{p_{k} }} + Dk^{ - s} \left[ {M\left( {\frac{{\left\| {Y_{k} ,Z} \right\|}}{{\rho_{2} }}} \right)} \right]^{{p_{k} }} \in F, \\ \end{aligned} $$

where *D* = max{1, 2^*H*−1^}. Thus *αX* + *βY* ∊ *F*(‖.,.‖, *M*, *p*, *s*) and completes the proof.

### **Theorem 2**

*F*(‖.,.‖, *M*, *p*, *s*) *is a paranormed space under the function g given by the* Eq. (1).

### *Proof*

Since *g*_*F*_ is a paranorm on *F*, by definition *g*(*X*) ≥ 0, ∀*X* ∊ *F*(‖.,.‖, *M*, *p*, *s*). Clearly, $\bar{g}(\theta ) = 0$.

Again, by property (N3) in the definition of 2-norm, *g*(−*X*) = *g*(*X*) holds for all *X* ∊ *F*(‖.,.‖, *M*, *p*, *s*).

Also, by taking *α* = *β* = 1 in the previous theorem and using the fact that *g*_*F*_ is monotone, we get *g*(*X* + *Y*) ≤ *g*(*X*) + *g*(*Y*) for *X* = (*X*_*k*_), *Y* = (*Y*_*k*_) ∊ *F*(‖.,.‖, *M*, *p*, *s*).

We are only left to show that *g* is continuous under scalar multiplication.

Let *λ* be any number. Then for some *ρ* > 0,

$$ \begin{aligned} g(\lambda X) & = {\text{inf}}\left\{ {\rho^{{p_{k} /T}} > 0: \left[ {g_{F} \left( {k^{ - s} \left[ {M\left( {\frac{{\left\| {\lambda X_{k} ,Z} \right\|}}{\rho }} \right)} \right]^{{p_{k} }} } \right)} \right]^{1/T} \le 1, \quad k = 1,2, \ldots } \right\} \\ & = {\text{inf}}\left\{ {\rho^{{p_{k} /T}} > 0: \left[ {g_{F} \left( {k^{ - s} \left[ {M\left( {\frac{{|\lambda |\left\| {X_{k} ,Z} \right\|}}{\rho }} \right)} \right]^{{p_{k} }} } \right)} \right]^{1/T} \le 1, \quad k = 1,2, \ldots } \right\} \\ \end{aligned} $$

Let *r* = *ρ*/|*λ*|. Then

$$ g(\lambda X) = {\text{inf}}\left\{ {(|\lambda |r)^{{p_{k} /T}} > 0: \left[ {g_{F} \left( {k^{ - s} \left[ {M\left( {\frac{{\left\| {X_{k} ,Z} \right\|}}{r}} \right)} \right]^{{p_{k} }} } \right)} \right]^{1/T} \le 1, \quad k = 1,2, \ldots } \right\} $$

Since $|\lambda |^{{p_{k} }} \le max(1, |\lambda |^{H} )$. So, $|\lambda |^{{p_{k} /T}} \le (max(1, |\lambda |^{H} ))^{1/T}$. Therefore, it converges to zero if *g*(*X*) converges to zero in *F*(‖.,.‖, *M*, *p*, *s*).

Now suppose *λ*_*n*_ → 0 as *n* → ∞ and let *X* = (*X*_*k*_) ∊ *F*(‖.,.‖, *M*, *p*, *s*).

Let *ɛ* > 0 be arbitrarily chosen and let *K* be a positive integer such that for some *ρ* > 0,

$$ g_{F} \left( {k^{ - s} \left[ {M\left( {\frac{{\left\| {X_{k} ,Z} \right\|}}{\rho }} \right)} \right]^{{p_{k} }} } \right) < \varepsilon /2, \quad {\text{for}}\; k > K $$

which implies for *k* > *N*,

$$ \left[ {g_{F} \left( {k^{ - s} \left[ {M\left( {\frac{{\left\| {X_{k} ,Z} \right\|}}{\rho }} \right)} \right]^{{p_{k} }} } \right)} \right]^{1/T} \le \varepsilon /2 $$

Let 0 < |*λ*| < 1, using convexity of *M* and the property (N3) of 2-norm, for *k* > *K* we get

$$ \begin{aligned} g_{F} \left( {k^{ - s} \left[ {M\left( {\frac{{\left\| {\lambda X_{k} ,Z} \right\|}}{\rho }} \right)} \right]^{{p_{k} }} } \right) & = g_{F} \left( {k^{ - s} \left[ {M\left( {|\lambda |\frac{{\left\| {X_{k} ,Z} \right\|}}{\rho }} \right)} \right]^{{p_{k} }} } \right) \\ & < g_{F} \left( {k^{ - s} \left[ {|\lambda |M\left( {\frac{{\left\| {X_{k} ,Z} \right\|}}{\rho }} \right)} \right]^{{p_{k} }} } \right) \\ & < g_{F} \left( {k^{ - s} \left[ {M\left( {\frac{{\left\| {X_{k} ,Z} \right\|}}{\rho }} \right)} \right]^{{p_{k} }} } \right) \\ & < (\varepsilon /2)^{T} \\ \end{aligned} $$

Since *M* is continuous everywhere in [0, ∞) and by the definition of *g*_*F*_, it follows that for *k* ≤ *K*

$$ \varphi (t) = g_{F} \left( {k^{ - s} \left[ {M\left( {\frac{{\left\| {tX_{k} ,Z} \right\|}}{\rho }} \right)} \right]^{{p_{k} }} } \right) $$

is continuous at 0.

So, there is 0 < *δ* < 1 such that |*φ*(*t*)| < *ɛ*/2 for 0 < *t* < *δ*. Let *L* be such that |*λ*_*n*_| < *δ* for *n* > *L*, then

$$ \left[ {g_{F} \left( {k^{ - s} \left[ {M\left( {\frac{{\left\| {\lambda_{n} X_{k} ,Z} \right\|}}{\rho }} \right)} \right]^{{p_{k} }} } \right)} \right]^{1/T} < \varepsilon /2 $$

for *n* > *L* and *k* ≤ *K*. Hence

$$ \left[ {g_{F} \left( {k^{ - s} \left[ {M\left( {\frac{{\left\| {\lambda_{n} X_{k} ,Z} \right\|}}{\rho }} \right)} \right]^{{p_{k} }} } \right)} \right]^{1/T} < \varepsilon $$

for *n* > *L* and for all *k*. Hence *λ*_*n*_ *X* → *θ* as *n* → ∞.

### **Theorem 3**

*Let the base space* (*N*, ‖.,.‖) *be a* 2-*Banach space. Then F*(‖.,.‖, *M*, *p*, *s*) *is a complete paranormed space under the paranorm g given by* (1), *where F is a K-space*.

### *Proof*

Let (*X* ^*i*^) be a Cauchy Sequence in *F*(‖.,.‖, *M*, *p*, *s*). Then *g*(*X* ^*i*^ − *X* ^*j*^) → 0 as *i*, *j* → ∞. For any given *ɛ* > 0, let *r* and *x*_0_ be such that $\frac{\varepsilon }{{rx_{0} }} > 0$ and $M(\frac{{rx_{0} }}{2}) \ge \mathop {sup}\nolimits_{k \ge 1} k^{{s/p_{k} }}$.

Now *g*(*X* ^*i*^ − *X* ^*j*^) → 0 as *i*, *j* → ∞ implies that there exist $N_{0} \in {\mathbb{N}}$ such that

$$ g(X^{i} - X^{j} ) < \frac{\varepsilon }{{rx_{0} }} \quad {\text{for}}\;{\text{all}}\; i,j \ge N_{0} $$

Then we have for *i*, *j* ≥ *N*_0_ such that for every *z* ∊ *N*,

$${\rm  inf}\left\{ {\rho^{{p_{k} /T}} > 0: \left[ {g_{F} \left( {k^{ - s} \left[ {M\left( {\frac{{\left\| {X_{k}^{i} - X_{k}^{j} ,Z} \right\|}}{\rho }} \right)} \right]^{{p_{k} }} } \right)} \right]^{1/T} \le 1, k = 1,2, \ldots } \right\} < \frac{\varepsilon }{{rx_{0} }} $$

Hence we have for every *z* ∊ *N*,

$$ g_{F} \left( {k^{ - s} \left[ {M\left( {\frac{{\left\| {X_{k}^{i} - X_{k}^{j} ,Z} \right\|}}{{g\left( {X^{i} - X^{j} } \right)}}} \right)} \right]^{{p_{k} }} } \right) \le 1,\quad {\text{for}}\,\,i, j \ge N_{0} $$

Since *F* is a *K*-space, *p*_*k*_ ≥ 0 and we can choose *s* suitably so that

$$ k^{ - s} \left[ {M\left( {\frac{{\left\| {X_{k}^{i} - X_{k}^{j} ,Z} \right\|}}{{g\left( {X^{i} - X^{j} } \right)}}} \right)} \right]^{{p_{k} }} \le 1 $$

for each *k* and for *i*, *j* ≥ *N*_0_ and *z* ∊ *N*.

Therefore,

$$ M\left( {\frac{{\left\| {X_{k}^{i} - X_{k}^{j} ,Z} \right\|}}{{g(X^{i} - X^{j} )}}} \right) \le k^{{s/p_{k} }} \le M\left( {\frac{{rx_{0} }}{2}} \right) $$

Thus we get

$$ \left\| {X_{k}^{i} - X_{k}^{j} ,Z} \right\| < \frac{\varepsilon }{{rx_{0} }}\frac{{rx_{0} }}{2} = \frac{\varepsilon }{2} $$

for each *k* and for *i*, *j* ≥ *N*_0_ and for every *z* ∊ *N*.

Therefore $\left( {X_{k}^{i} } \right)$ becomes a Cauchy sequence in *N*. Since (*N*, ‖.,.‖) is complete, there exist *X* = (*X*_*k*_) ∊ *N* such that $X_{k}^{i} \to X_{k}$ as *i* → ∞ for each *k*. Since *M* is continuous it shows that

$$ M\left( {\frac{{\left\| {X_{k} - X_{k}^{j} ,Z} \right\|}}{\rho }} \right) \to 0 \quad {\text{as}}\; i \to \infty $$

for each *k*, *z* ∊ *N* and for some *ρ* > 0. Consequently,

$$ k^{ - s} \left[ {M\left( {\frac{{\left\| {X_{k} - X_{k}^{j} ,Z} \right\|}}{\rho }} \right)} \right]^{{p_{k} }} \to 0 \quad {\text{as}}\; i \to \infty $$

for each *k*, *z* ∊ *N* and for some *ρ* > 0.

Let

$$ \alpha_{k}^{j} = k^{ - s} \left[ {M\left( {\frac{{\left\| {X_{k} - X_{k}^{j} ,Z} \right\|}}{\rho }} \right)} \right]^{{p_{k} }} $$

Then since *M* is non-decreasing, by suitable choice of *δ* (depending on *j* and *k*),

$$ \alpha_{k}^{j} < \delta k^{ - s} \left[ {M\left( {\frac{{\left\| {X_{k}^{j} ,Z} \right\|}}{\rho }} \right)} \right]^{{p_{k} }} $$

where 0 < *δ* < 1. Since *F* is normal, it follows that (*α* ^*i*^) ∊ *F* for each *i*. Also $\alpha_{k}^{i} \to 0$ as *i* → ∞ implies that *g*_*F*_(*α* ^i^) → 0 as *i* → ∞. Hence *X* ^*i*^ → ^*g*^ *X* as *i* → ∞ in *F*(‖.,.‖, *M*, *p*, *s*). Again

$$ \begin{aligned} k^{ - s} \left[ {M\left( {\frac{{\left\| {X_{k} , Z} \right\|}}{\rho }} \right)} \right]^{{p_{k} }} & = k^{ - s} \left[ {M\left( {\frac{{\left\| {X_{k}^{i} + \left( {X_{k} - X_{k}^{i} } \right), Z} \right\|}}{\rho }} \right)} \right]^{{p_{k} }} \\ & \le Dk^{ - s} \left[ {M\left( {\frac{{\left\| {X_{k}^{i} , Z} \right\|}}{\rho }} \right)} \right]^{{p_{k} }} + D\alpha_{k}^{i} ({\text{where}}\; D = max\{ 1,2^{H - 1} \} ) \\ & \le D(1 + \delta )k^{ - s} \left[ {M\left( {\frac{{\left\| {X_{k}^{i} , Z} \right\|}}{\rho }} \right)} \right]^{{p_{k} }} \\ \end{aligned} $$

Since (*X* ^*i*^) ∊ *F*(‖.,.‖, *M*, *p*, *s*) and *F* is a normal space, it seems that *X* = (*X*_*k*_) ∊ *F*(‖.,.‖, *M*, *p*, *s*). Hence it is complete.

### **Theorem 4**

*F*(‖.,.‖, *M*, *p*, *s*) *is a K-space if F is a K-space.*

### *Proof*

Let us define a mapping

$$ P_{n} :F\left( {\left\| {.,.} \right\|,M,p,s} \right) \to N $$

by $P_{n} (X) = X_{n} , \forall n \in {\mathbb{N}}$. To show *P*_*n*_ is continuous.

Let (*X* ^*m*^) be a sequence in *F*(‖.,.‖, *M*, *p*, *s*) such that *X* ^*m*^ → ^*g*^0 as *m* → ∞. Then for some suitable choice of *ρ* > 0,

$$ \left[ {g_{F} \left( {k^{ - s} \left[ {M\left( {\frac{{\left\| {X_{k}^{m} ,Z} \right\|}}{\rho }} \right)} \right]^{{p_{k} }} } \right)} \right]^{1/T} \to 0 \quad {\text{as}}\; m \to \infty $$

Since *F* is a *K*-space, this implies that for each *k* and as *m* tending to ∞,

$$ k^{ - s} \left[ {M\left( {\frac{{\left\| {X_{k}^{m} ,Z} \right\|}}{\rho }} \right)} \right]^{{p_{k} }} \to 0 $$

for some *ρ* > 0. Since *M* is an Orlicz function, it follows that

$$ \left\| {X_{k}^{m} ,Z} \right\| \to 0 \quad {\text{as}}\; m \to \infty . $$

Consequently, *X* ^*m*^ → 0 in *N*. Hence the proof.

## Relationship results

In this section, we shall investigate the relationship among the spaces defined in second section and their possible variants under different conditions.

In the next two results, we shall shows how the addition and composition of two different Orlicz functions effect the spaces in term of their relationship of size.

### **Theorem 5**

*Let M*_1_ *and M*_2_ *be two Orlicz functions. Then*

$$ F\left( {\left\| {.,.} \right\|,M_{1} ,p,s} \right) \cap F\left( {\left\| {.,.} \right\|,M_{2} ,p,s} \right) \subseteq F\left( {\left\| {.,.} \right\|,M_{1} + M_{2} ,p,s} \right), $$

*where F is a normal sequence space.*

### *Proof*

Let *X* = (*X*_*k*_) ∊ *F*(‖.,.‖, *M*_1_, *p*, *s*) ∩ *F*(‖.,.‖, *M*_2_, *p*, *s*). Then we can choose *ρ*_1_, *ρ*_2_ > 0 such that

$$ \left( {k^{ - s} \left[ {M_{1} \left( {\frac{{\left\| {X_{k} ,Z} \right\|}}{{\rho_{1} }}} \right)} \right]^{{p_{k} }} } \right) \in F $$

and

$$ \left( {k^{ - s} \left[ {M_{2} \left( {\frac{{\left\| {X_{k} ,Z} \right\|}}{{\rho_{2} }}} \right)} \right]^{{p_{k} }} } \right) \in F $$

Let us choose *ρ* = max(*ρ*_1_, *ρ*_2_). Then

$$ \begin{aligned}  k^{ - s} \left[ {\left( {M_{1} + M_{2} } \right)\left( {\frac{{\left\| {X_{k} ,Z} \right\|}}{\rho }} \right)} \right]^{{p_{k} }} & \le k^{ - s} D\left\{ {\left[ {M_{1} \left( {\frac{{\left\| {X_{k} ,Z} \right\|}}{{\rho_{1} }}} \right)} \right]^{{p_{k} }} } \right. \\ & \quad \left. { + \left[ {M_{2} \left( {\frac{{\left\| {X_{k} ,Z} \right\|}}{{\rho_{2} }}} \right)} \right]^{{p_{k} }} } \right\} \in F,\,\,\,\,\,{\text{where}}\,\,D = { \hbox{max} }\left( {1, 2^{H - 1} } \right) \\ \end{aligned} $$

Now the proof follows immediately as *F* being normal.

### **Theorem 6**

*Let M*_1_ *and M*_2_ *be Orlicz functions satisfying* Δ_2_ *-condition. Then we have the following inclusion*

$$ F\left( {\left\| {.,.} \right\|, M_{1} , p, s} \right) \subseteq F(\left\| {.,.} \right\|, M_{2} \circ M_{1} , p, s)\,{\text{if}}\,s > 1. $$

### *Proof*

Let *X* = (*X*_*k*_) ∊ *F*(‖.,.‖, M_1_, p, s). Since *M*_2_ is continuous from the right at 0, there exists 0 < *η* < 1 such that for any arbitrary *ɛ* > 0, *M*_2_(*t*) < *ɛ* whenever 0 ≤ *t* ≤ *η*.

Let us define the sets

$$ N_{1} = \left\{ {k \in {\mathbb{N}}: M_{1} \left( {\frac{{\left\| {X_{k} ,Z} \right\|}}{\rho }} \right) \le \eta } \right\} $$

$$ N_{2} = \left\{ {k \in {\mathbb{N}}: M_{1} \left( {\frac{{\left\| {X_{k} ,Z} \right\|}}{\rho }} \right) > \eta } \right\} $$

for some *ρ* > 0.

If *k* ∊ *N*_2_,

$$ \begin{aligned} M_{1} \left( {\frac{{\left\| {X_{k} ,Z} \right\|}}{\rho }} \right) & < \frac{1}{\eta }M_{1} \left( {\frac{{\left\| {X_{k} ,Z} \right\|}}{\rho }} \right) \\ & < 1 + \left[ {\frac{1}{\eta }M_{1} \left( {\frac{{\left\| {X_{k} ,Z} \right\|}}{\rho }} \right)} \right] \\ \end{aligned} $$

Since *M*_2_ is non-decreasing and convex it follows that

$$ \begin{aligned} M_{2} \left[ {M_{1} \left( {\frac{{\left\| {X_{k} ,Z} \right\|}}{\rho }} \right)} \right] & < M_{2} \left[ {1 + \frac{1}{\eta }M_{1} \left( {\frac{{\left\| {X_{k} ,Z} \right\|}}{\rho }} \right)} \right] \\ & < \frac{1}{2}M_{2} (2) + \frac{1}{2}M_{2} \left[ {2\frac{1}{\eta }M_{1} \left( {\frac{{\left\| {X_{k} ,Z} \right\|}}{\rho }} \right)} \right] \\ \end{aligned} $$

Again since *M*_2_ satisfies Δ_2_-condition, we have

$$ \begin{aligned} M_{2} \left[ {M_{1} \left( {\frac{{\left\| {X_{k} ,Z} \right\|}}{\rho }} \right)} \right] & < \frac{1}{2}L\left[ {\frac{1}{\eta }M_{1} \left( {\frac{{\left\| {X_{k} ,Z} \right\|}}{\rho }} \right)} \right]M_{2} (2) \\ & \quad + \frac{1}{2}L\left[ {\frac{1}{\eta }M_{1} \left( {\frac{{\left\| {X_{k} ,Z} \right\|}}{\rho }} \right)} \right]M_{2} (2) \\ & = L\eta^{ - 1} M_{2} (2)M_{1} \left( {\frac{{\left\| {X_{k} ,Z} \right\|}}{\rho }} \right) \\ \end{aligned} $$

So,

2 $$ k^{ - s} \left[ {M_{2} \left( {M_{1} \left( {\frac{{\left\| {X_{k} ,Z} \right\|}}{\rho }} \right)} \right)} \right]^{{p_{k} }} \le k^{ - s} D_{1} \left[ {M_{1} \left( {\frac{{\left\| {X_{k} ,Z} \right\|}}{\rho }} \right)} \right]^{{p_{k} }} $$

where *D*_1_ = max{1, [*Lη* ^−1^ *M*_2_(2)]^*H*^}.

For, *k* ∊ *N*_1_,

$$ M_{1} \left( {\frac{{\left\| {X_{k} ,Z} \right\|}}{\rho }} \right) \le \eta \Rightarrow M_{2} \left[ {M_{1} \left( {\frac{{\left\| {X_{k} ,Z} \right\|}}{\rho }} \right)} \right] < \varepsilon $$

and therefore,

3 $$ k^{ - s} \left[ {M_{2} \left[ {M_{1} \left( {\frac{{\left\| {X_{k} ,Z} \right\|}}{\rho }} \right)} \right]} \right]^{{p_{k} }} < k^{ - s} [\varepsilon ]^{H} $$

Hence from (2) and (3) we have

$$ \begin{aligned} & k^{ - s} \left[ {M_{2} \left[ {M_{1} \left( {\frac{{\left\| {X_{k} ,Z} \right\|}}{\rho }} \right)} \right]} \right]^{{p_{k} }} \\ & \quad \le k^{ - s} [\varepsilon ]^{H} + k^{ - s} D_{1} \left[ {M_{1} \left( {\frac{{\left\| {X_{k} ,Z} \right\|}}{\rho }} \right)} \right]^{{p_{k} }} \in F \\ \end{aligned} $$

for all *k*. Then the proof follows by the normality of *F*.

We have the well known inclusion *c*_0_ ⊂ *c* ⊂ *ℓ*_∞_. The following result shows that if *F* is replaced by these three spaces, the corresponding extended versions also preserve this inclusion.

### **Theorem 7**

*Let M be an Orlicz function. Then*

$$ c_{0} \left( {\left\| {.,.} \right\|,M,p,s} \right) \subset c\left( {\left\| {.,.} \right\|,M,p,s} \right) \subset \ell_{\infty } \left( {\left\| {.,.} \right\|,M,p,s} \right) $$

### *Proof*

The first inclusion follows immediately from the definitions. For second inclusion, let *X* = (*X*_*k*_) ∊ *c*(‖.,.‖, *M*, *p*, *s*). Then for some *ρ* = 2*η* > 0, we have

$$ \begin{aligned} & k^{ - s} \left[ {M\left( {\frac{{\left\| {X_{k} ,Z} \right\|}}{\rho }} \right)} \right]^{{p_{k} }} \\ & \quad = k^{ - s} \left[ {M\left( {\frac{{\left\| {X_{k} - L + L,Z} \right\|}}{\rho }} \right)} \right]^{{p_{k} }} \\ & \quad \le k^{ - s} \left[ {M\left( {\frac{{\left\| {X_{k} - L,Z} \right\| + \left\| {L,Z} \right\|}}{\rho }} \right)} \right]^{{p_{k} }} \\ & \quad \le k^{ - s} D\left[ {M\left( {\frac{{\left\| {X_{k} - L,Z} \right\|}}{\eta }} \right)} \right]^{{p_{k} }} + k^{ - s} D\left[ {M\left( {\frac{{\left\| {L,Z} \right\|}}{\eta }} \right)} \right]^{{p_{k} }} \\ & \quad \le k^{ - s} D\left[ {M\left( {\frac{{\left\| {X_{k} - L,Z} \right\|}}{\eta }} \right)} \right]^{{p_{k} }} + k^{ - s} Dmax\left\{ {1,\left[ {M\left( {\frac{{\left\| {L,Z} \right\|}}{\eta }} \right)} \right]^{H} } \right\} \\ \end{aligned} $$

Thus *X* = (*X*_*k*_) ∊ *ℓ*_∞_ (‖.,.‖, *M*, *p*, *s*).

Our next result is to examine the effect of the parameter p on the relationships of some spaces.

### **Theorem 8**

*Let M be a Orlicz function. Then*

(i) *If* 0 < inf *p*_*k*_ ≤ *p*_*k*_ < 1 *then c*_0_(‖.,.‖, *M*, *s*) ⊂ *c*_0_(‖.,.‖, *M*, *p*, *s*);
(ii) *If* 1 ≤ *p*_*k*_ ≤ supp_*k*_ < ∞, *then c*_0_(‖.,.‖, *M*, *p*, *s*) ⊂ *c*_0_(‖.,.‖, *M*, *s*).

### *Proof*

(*i*) Let *X* = (*X*_*k*_) ∊ *c*_0_(‖.,.‖, *M*, *s*). Since 0 < inf *p*_*k*_ ≤ *p*_*k*_ < 1 the proof follows from the following inequality

$$ \left[ {M\left( {\frac{{\left\| {X_{k} ,Z} \right\|}}{\rho }} \right)} \right]^{{p_{k} }} \le M\left( {\frac{{\left\| {X_{k} ,Z} \right\|}}{\rho }} \right). $$

### *Proof*

(*ii*) Let 1 ≤ *p*_*k*_ ≤ supp_*k*_ < ∞ and *X* = (*X*_*k*_) ∊ *c*_0_(‖.,.‖, *M*, *p*, *s*). Then for each 0 < *ɛ* < 1 there exists a positive integer *L* such that

$$ k^{ - s} \left[ {M\left( {\frac{{\left\| {X_{k} ,Z} \right\|}}{\rho }} \right)} \right]^{{p_{k} }} \le \varepsilon < 1\quad \forall k \ge L $$

Since 1 ≤ *p*_*k*_ ≤ supp_*k*_ < ∞, the proof follows from the following inequality

$$ k^{ - s} \left[ {M\left( {\frac{{\left\| {X_{k} ,Z} \right\|}}{\rho }} \right)} \right] \le k^{ - s} \left[ {M\left( {\frac{{\left\| {X_{k} ,Z} \right\|}}{\rho }} \right)} \right]^{{p_{k} }} $$

The Orlicz functions are often used to extend sets of sequences in order to study algebraic and topological properties using the rich properties of Orlicz functions. The following result gives us a equality connection of composite Orlicz sequence spaces with those of spaces defined without Orlicz function.

### **Theorem 9**

*Let M be a Orlicz function satisfying* Δ_2_ *-condition and* 0 *<* *A*_1_ *≤* *M*(*t*)*/t* *≤* *A*_2_ *for t* > 0*, where A*_1_ *and A*_2_ *are constants. Then*

$$ F\left( {\left\| {.,.} \right\|, M^{r} , p, s} \right) = F\left( {\left\| {.,.} \right\|, p, s} \right),\quad r\,{\text{is}}\,{\text{a}}\,{\text{positive}}\,{\text{integer}}. $$

### *Proof*

Let us take the left part of the inequality. Then we get

$$ t \le \frac{1}{{A_{1} }}M(t) $$

So, we have

4 $$ t \le \frac{1}{{A_{1} }}M(t) < \left( {1 + \left[ {\frac{1}{{A_{1} }}} \right]} \right)M(t) $$

Since *M* satisfies Δ_2_-condition,

5 $$ M(t) < M\left[ {\left( {1 + \left[ {\frac{1}{{A_{1} }}} \right]} \right)M(t)} \right] \le L\left( {1 + \left[ {\frac{1}{{A_{1} }}} \right]} \right)^{{\log_{2} L}} M^{2} (t), $$

for some constant *L* > 0. From (4) and (5) we get

$$ t < L\left( {1 + \left[ {\frac{1}{{A_{1} }}} \right]} \right)^{{1 + \log_{2} L}} M^{2} (t) $$

Hence, after *r* steps we get

6 $$ t < L^{{r - 1 + (r - 2)\log_{2} L + (r - 3)\left( {\log_{2} L} \right)^{2} + (r - 4)\left( {\log_{2} L} \right)^{3} + \cdots + \left( {\log_{2} L} \right)^{r - 2} }} \left( {1 + \left[ {\frac{1}{{A_{1} }}} \right]} \right)^{{1 + \log_{2} L + \left( {\log_{2} L} \right)^{2} + \left( {\log_{2} L} \right)^{3} + \left( {\log_{2} L} \right)^{4} + \cdots + \left( {\log_{2} L} \right)^{r - 1} }} M^{r} (t) $$

Let *X* = (*X*_*k*_) ∊ *F*(‖.,.‖, *M* ^*r*^, *p*, *s*). Then $$ \left( {k^{ - s} \left[ {M^{r} \left( {\frac{{\left\| {X_{k} ,Z} \right\|}}{\rho }} \right)} \right]^{{p_{k} }} } \right) \in F $$.

On taking $t = \frac{{\left\| {X_{k} ,Z} \right\|}}{\rho }$ in (6), we get

$$ k^{ - s} \left[ {\frac{{\left\| {X_{k} ,Z} \right\|}}{\rho }} \right]^{{p_{k} }} < \left\{ {L^{{r - 1 + (r - 2)\log_{2} L + (r - 3)\left( {\log_{2} L} \right)^{2} + (r - 4)\left( {\log_{2} L} \right)^{3} + \cdots + \left( {\log_{2} L} \right)^{r - 2} }} \left( {1 + \left[ {\frac{1}{{A_{1} }}} \right]} \right)^{{1 + \log_{2} L + \left( {\log_{2} L} \right)^{2} + \left( {\log_{2} L} \right)^{3} + \left( {\log_{2} L} \right)^{4} + \cdots + \left( {\log_{2} L} \right)^{r - 1} }} } \right\}^{H} k^{ - s} \left[ {M^{r} \left( {\frac{{\left\| {X_{k} ,Z} \right\|}}{\rho }} \right)} \right]^{{p_{k} }} $$

Since *F* is normal, it follows that $(k^{ - s} [\left\| {X_{k} ,Z)} \right\|]^{{p_{k} }} ) \in F$. Consequently, *X* = (*X*_*k*_) ∊ *F*(‖.,.‖, *p*, *s*).

Next, let us consider the right part of the inequality. Then we get

$$ M(t) \le A_{2} t < \left( {1 + \left[ {A_{2} } \right]} \right)t $$

which implies

7 $$ M^{r} \left( t \right) < L^{{1 + \log_{2} L + \left( {\log_{2} L} \right)^{2} + \cdots + \left( {\log_{2} L} \right)^{r - 2} }} \left( {1 + \left[ {A_{2} } \right]} \right)^{{1 + \log_{2} L + \left( {\log_{2} L} \right)^{2} + \left( {\log_{2} L} \right)^{3} + \cdots + \left( {\log_{2} L} \right)^{r - 1} }} t, $$

for some constant *L* > 0.

Let *X* = (*X*_*k*_) ∊ *F*(‖.,.‖, *p*, *s*). Then

$$ \left( {k^{ - s} \left[ {\left\| {X_{k} ,Z} \right\|} \right]^{{p_{k} }} } \right) \in F $$

From the inequality (7) and proceeding similarly as in the previous part we have

$$ k^{ - s} \left[ {M^{r} \left( {\frac{{\left\| {X_{k} ,Z} \right\|}}{\rho }} \right)} \right]^{{p_{k} }} < \left[ {L^{{1 + \log_{2} L + (\log_{2} L)^{2} + \cdots + ({ \log }_{2} L)^{r - 2} }} \left( {1 + [A_{2} ]} \right)^{{1 + \log_{2} L + (\log_{2} L)^{2} + (\log_{2} L)^{3} + \cdots + (\log_{2} L)^{r - 1} }} } \right]^{H} k^{ - s} \left[ {\frac{{\left\| {X_{k} ,Z} \right\|}}{\rho }} \right]^{{p_{k} }} \in F $$

for each $k \in {\mathbb{N}}$. Hence *X* = (*X*_*k*_) ∊ *F*(‖.,.‖, *M* ^*r*^, *p*, *s*), *F* being normal.

In composite Orlicz sequence spaces, the following result gives a connection between such spaces which depend on the number of participating Orlicz functions and satisfying certain condition.

### **Theorem 10**

*Let M be an Orlicz function satisfying* Δ_2_ *-condition and M*(*t*)*/t* ≤ *A for t* *≥* 0*, where A is a constant. If* $r, n \in {\mathbb{N}}$ such that *r* > *n* then

$$ F\left( {\left\| {.,.} \right\|,M^{n} ,p,s} \right) \subseteq F\left( {\left\| {.,.} \right\|,M^{r} ,p,s} \right) $$

### *Proof*

Let *r* − *n* = *ℓ* > 0. Now

$$ M(t) \le At < (1 + [A])t $$

Since *M* satisfying Δ_2_-condition, we have after *r*th step,

$$ M^{r} \left( t \right) < L^{{1 + \log_{2} L + \left( {\log_{2} L} \right)^{2} + \cdots + \left( {\log_{2} L} \right)^{r - 2} }} \left( {1 + \left[ A \right]} \right)^{{1 + \log_{2} L + \left( {\log_{2} L} \right)^{2} + \left( {\log_{2} L} \right)^{3} + \cdots + \left( {\log_{2} L} \right)^{r - 1} }} t, $$

for some constant *L* > 0.

Therefore

8 $$ \begin{aligned} M^{r} (t) & = M^{n + l} (t) = M^{n} [M^{l} (t)] \\ & < M^{n} \left[ {L^{{1 + \log_{2} L + \left( {\log_{2} L} \right)^{2} + \cdots + \left( {\log_{2} L} \right)^{l - 2} }} \left( {1 + \left[ A \right]} \right)^{{1 + \log_{2} L + \left( {\log_{2} L} \right)^{2} + \left( {\log_{2} L} \right)^{3} + \cdots + \left( {\log_{2} L} \right)^{l - 1} }} t} \right] \\ & \le \left( {L^{{1 + \log_{2} L + \left( {\log_{2} L} \right)^{2} + \left( {\log_{2} L} \right)^{3} + \cdots + \left( {\log_{2} L} \right)^{l + (n - 2)} }} \left( {1 + \left[ A \right]} \right)^{{\left( {\log_{2} L} \right)^{n} + \cdots + \left( {\log_{2} L} \right)^{l + (n - 1)} }} } \right)M^{n} \left( t \right) \\ \end{aligned} $$

Let *X* = (*X*_*k*_) ∊ *F*(‖.,.‖, *M* ^*n*^, *p*, *s*). Then

$$ \left( {k^{ - s} \left[ {M^{n} \left( {\frac{{\left\| {X_{k} ,Z} \right\|}}{\rho }} \right)} \right]^{{p_{k} }} } \right) \in F $$

Replacing *t* by ‖*X*_*k*_, *Z*‖/*ρ* on both sides of (8) we get

$$ M^{r} \left( {\frac{{\left\| {X_{k} ,Z} \right\|}}{\rho }} \right) \le \left( {L^{{1 + \log_{2} L + \left( {\log_{2} L} \right)^{2} + \left( {\log_{2} L} \right)^{3} + \cdots + \left( {\log_{2} L} \right)^{l + (n - 2)} }} \left( {1 + \left[ A \right]} \right)^{{\left( {\log_{2} L} \right)^{n} + \cdots + \left( {\log_{2} L} \right)^{l + (n - 1)} }} } \right)M^{n} \left( {\frac{{\left\| {X_{k} ,Z} \right\|}}{\rho }} \right) $$

This implies

$$ k^{ - s} \left[ {M^{r} \left( {\frac{{\left\| {X_{k} ,Z} \right\|}}{\rho }} \right)} \right]^{{p_{k} }} \le \left( {L^{{1 + \log_{2} L + \left( {\log_{2} L} \right)^{2} + \left( {\log_{2} L} \right)^{3} + \cdots + \left( {\log_{2} L} \right)^{l + (n - 2)} }} \left( {1 + \left[ A \right]} \right)^{{\left( {\log_{2} L} \right)^{n} + \cdots + \left( {\log_{2} L} \right)^{l + (n - 1)} }} } \right)^{H} k^{ - s} \left[ {M^{n} \left( {\frac{{\left\| {X_{k} ,Z} \right\|}}{\rho }} \right)} \right]^{{p_{k} }} \in F $$

It follows that *X* = (*X*_*k*_) ∊ *F*(‖.,.‖, *M* ^*r*^, *p*, *s*) due to the normality of *F*. Hence the proof.

## Further properties

In this section, we shall investigate essentially few more properties. These properties may influence the readers to study further such spaces for several other algebraic and topological behaviours including those of dual spaces.

The space *F*(‖.,.‖, *M*, *p*, *s*) is not convergence free in general. In order to establish it, it is easy to construct an example. Hence we have the following result.

### *Remark 1*

The space *F*(‖.,.‖, *M*, *p*, *s*) is not convergence free in general.

### *Proof*

Consider $F = \ell_{\infty } , s = 0, p_{k} = 1,\quad {\text{for}}\;{\text{each}}\,k \in N\; ,\,M(x) = x^{2} , \quad{\text{for}}\;{\text{all}}\; x \in [0,\infty ),$ and let us take the 2-norm ‖*x*, *y*‖ = sup_*i*∊*N*_ sup_*j*∊*N*_|*x*_*i*_ *y*_*j*_ − *x*_*j*_ *y*_*i*_|, where *x* = (*x*_1_, *x*_2_, …) and *y* = (*y*_1_, *y*_2_, …) ∊ *ℓ*_∞_.

Let us take (*X*_*k*_) ∊ *F*(‖.,.‖, *M*, *p*, *s*) defined as follows:

$$ {\text{For}}\,k\,{\text{even}},\,X_{k} = \frac{1}{k + 1} $$

and for *k* odd, *X*_*k*_ = 0.

Let us define a sequence (*Y*_*k*_) as follows:

$$ {\text{For}}\,k\,{\text{odd}}, \, Y_{k} = 0\quad {\text{and}}\quad {\text{for}}\,k\,{\text{even}},\,Y_{k} = k + 1 $$

Then *X*_*k*_ = 0 implies *Y*_*k*_ = 0, but (*Y*_*k*_) ∉ *F*(‖.,.‖, *M*, *p*, *s*).

However, the space *F*(‖.,.‖, *M*, *p*, *s*) is solid and symmetric in general. The following two results establish our claim with proof.

### **Theorem 11**

*The space F*(‖.,.‖, *M*, *p*, *s*) *is solid(normal) in general.*

### *Proof*

Let *X* = (*X*_*k*_) ∊ *F*(‖.,.‖, *M*, *p*, *s*), and *Y* = (*Y*_*k*_) be such that

$$ \left\| {Y_{k} , Z} \right\| \le \left\| {X_{k} , Z} \right\|\quad {\text{for}}\,{\text{every}}\,Z \in N. $$

Since *M* is non-decreasing,

$$ k^{ - s} \left[ {M\left( {\frac{{\left\| {Y_{k} , Z} \right\|}}{\rho }} \right)} \right]^{{p_{k} }} \le k^{ - s} \left[ {M\left( {\frac{{\left\| {X_{k} , Z} \right\|}}{\rho }} \right)} \right]^{{p_{k} }} \in F $$

for some *ρ* > 0. Hence *Y* = (*Y*_*k*_) ∊ *F*(‖.,.‖, *M*, *p*, *s*), since *F* is normal and the space is solid.

### **Theorem 12**

*The space F*(‖.,.‖, *M*, *p*, *s*) *is symmetric in general.*

### *Proof*

Let *X* = (*X*_*k*_) ∊ *F*(‖.,.‖, *M*, *p*, *s*), and $Y = (Y_{{m_{k} }} )$ be an arrangement of the sequence (*X*_*k*_) such that $X_{k} = Y_{{m_{k} }}$ for each *k* ∊ *N*. Then

$$ \left( {k^{ - s} \left[ {M\left( {\frac{{\left\| {Y_{{m_{k} }} , Z} \right\|}}{\rho }} \right)} \right]^{{p_{k} }} } \right) = \left( {k^{ - s} \left[ {M\left( {\frac{{\left\| {X_{k} , Z} \right\|}}{\rho }} \right)} \right]^{{p_{k} }} } \right) \in F $$

Hence these spaces are symmetric in general.

There is a close connection between Banach spaces and 2-Banach spaces. Now we shall try to reflect this connection in our definition of the spaces as well as in the completeness result.

Consider the norm ‖.‖ defined on a linear 2-normed space (*X*, ‖.,.‖) by the function

$$ \left\| a \right\| = \left\| {a,y} \right\| + \left\| {a,z} \right\| $$

for any fixed *y*, *z* ∊ *X* and ‖*y*, *z*‖ ≠ 0. Then the function ‖.‖ is a norm on *X* (Freese and Cho 2001).

We recall the following result and for details, we refer to (Freese and Cho 2001).

### **Proposition 13**

If (*X*, ‖.,.‖) is a linear 2-normed space possessing Property (*K*) (Freese and Cho 2001, p. 16) and having a norm defined on it by ‖*a*‖ = ‖a*, *a*‖ + ‖b*, *a*‖ for a* and b* in *X* such that ‖a*, b*‖ ≠ 0, then *X* is a 2-Banach space if and only if *X* is a Banach space relative to this norm.

For the sake of comparison between natural norm and the norm obtain from 2-norm as described above, we shall call the later as derived 1-norm or simply derived norm.

Using this concept of derived 1-norm, we can redefine our sets over derived 1-norm instead of 2-norm and we will get the similar results of this paper. If the 2-normed space (*N*, ‖.,.‖) possesses the Property (*K*), we can modify our completeness result as follows.

### **Proposition 14**

Let the 2-normed space (*N*, ‖.,.‖) possesses the Property (*K*). Then *F*(‖.,.‖, *M*, *p*, *s*) is a complete paranormed space under the paranorm *g* given by (1), where *F* is a *K*-space provided *N* is a Banach space.

## Conclusions

1. This new generalized class of Orlicz paranormed sequence space unifies many basic sequence spaces introduced by earlier authors;
2. The topological structure and different topological properties of this space characterize the general topological behavior of many sequence spaces introduced by earlier authors;
3. The inclusion relations between various spaces of sequences signify that some of the sequence spaces become identical or can be embedded to some other sequence spaces under certain conditions;
4. Some properties of the spaces investigated in the last section may attract further study on other aspects of such spaces.
5. Various results on 2-normed spaces may also be used to study several other convergence and completeness related properties of the spaces.
